# The Tension Between Cognitive and Regulatory Flexibility and Their Associations With Current and Lifetime PTSD Symptoms

**DOI:** 10.3389/fpsyg.2021.615289

**Published:** 2021-02-26

**Authors:** Shilat Haim-Nachum, Einat Levy-Gigi

**Affiliations:** ^1^School of Education, Bar-Ilan University, Ramat Gan, Israel; ^2^The Gonda Multidisciplinary Brain Research Center, Bar-Ilan University, Ramat Gan, Israel

**Keywords:** cognitive flexibility, regulatory flexibility, lifetime PTSD, current PTSD, trauma exposure

## Abstract

In recent years, researchers have tried to unpack the meaning of the term flexibility and test how different constructs of flexibility are associated with various psychopathologies. For example, it is apparent that high levels of flexibility allow individuals to adaptively cope and avoid psychopathology following traumatic events, but the precise nature of this flexibility is ambiguous. In this study we focus on two central constructs: *cognitive flexibility* – the ability to recognize and implement possible responses to a situation– and *regulatory flexibility* – the ability to modulate emotional expression and experience across situations. We aim to explore the connection between cognitive and regulatory flexibility and evaluate their relative effect on PTSD symptoms. Trauma-exposed college students (*N* = 109, *M* age = 25.31, *SD* = 4.59) were assessed for cognitive and regulatory flexibility and current and lifetime PTSD symptoms. We predicted and found a relatively weak, yet significant, overlap between participants’ cognitive and regulatory flexibility. Importantly, while both cognitive and regulatory flexibility were associated with lifetime PTSD symptoms, only cognitive flexibility was associated with current PTSD symptoms. The findings illuminate the possible value of differentiating between constructs of flexibility in predicting short and long-term effects of traumatic exposure and may pave the ground for developing personalized intervention methods.

## The Tension Between Cognitive and Regulatory Flexibility: Cognitive Flexibility Is More Strongly Associated With Current PTSD Symptoms

In recent years, the ability to function in a flexible manner has been widely studied and is considered one of the most important mechanisms associated with resilience and well-being (Bonanno et al., 2004; Bonanno and Burton, 2013; Koole et al., 2015; Wersebe et al., 2018; Gentili et al., 2019). Most studies referred to flexibility as a broad construct (for review, see, Kashdan and Rottenberg, 2010); lately, however, researchers have begun to more narrowly examine distinct flexibility constructs, illustrating the theoretical and clinical utility of each. These include coping flexibility (Kato, 2012; Cheng et al., 2014), affective flexibility (Zhu and Bonanno, 2017), expressive flexibility (Westphal et al., 2010), emotional flexibility (Fu et al., 2018), psychological flexibility (Whiting et al., 2017), and behavioral flexibility (Tei et al., 2017). The results of these studies may suggest that being adept at one type of flexibility does not necessarily indicate proficiency in another. Moreover, the relationship between different constructs of flexibility may be complex and multifaceted, highlighting the need for a more specific examination.

However, such an examination is complicated since there are definitional overlaps, with different terms referring to the same construct of flexibility; for example, research on coping and emotion regulation uses *coping flexibility, affective flexibility* and *emotion regulation flexibility* to depict individuals’ ability to modulate subjective feeling and behaviors while utilizing emotion regulation strategies across stressful situations (Bonanno et al., 2011; Cheng et al., 2014; Zhu and Bonanno, 2017; Southward et al., 2018). Furthermore, individuals’ ability to regulate their emotions in accordance with contextual demands is sometimes labeled expressive flexibility (Bonanno et al., 2004) and sometimes cognitive flexibility (Moore and Malinowski, 2009; Gabrys et al., 2018).

In the current study, we focus on two main constructs which have received substantial attention in the literature, namely, cognitive, and regulatory flexibility. Understanding the interplay between these two constructs is especially important due to their significant associations with various psychopathologies, including depression, anxiety, bipolar, and eating disorders (for studies on cognitive flexibility, see, O’Donnell et al., 2017; Perpiñá et al., 2017; Gabrys et al., 2018; for studies on regulatory flexibility, see, Southward and Cheavens, 2017; Southward et al., 2018). The ultimate goal of the present study is to define and explore the relationship between cognitive and regulatory flexibility, as well as their link to symptoms of post-traumatic stress disorder (PTSD). We refer to *cognitive flexibility* as the ability to recognize multiple possible responses to a situation and to make an adaptive choice (Martin and Rubin, 1995) and to *regulatory flexibility* as an individual’s ability to modulate emotional experience, as well as the perceived ability to use different coping strategies in a way that suits contextual demands (Bonanno and Burton, 2013). Our *first aim* is to test whether these constructs of flexibility represent one unified skill or two independent abilities that at times overlap. While it is generally accepted that cognition and emotion are highly interdependent processes (Pessoa, 2008; Dolcos et al., 2011), the extent of the overlap between cognitive and regulatory *flexibility* is less clear. Given that both constructs refer to flexibility, they are likely to share certain features. We therefore expected to find a significant, yet relatively weak connection between the two.

Our *second aim* is to examine the independent and relative effects of cognitive and regulatory flexibility on PTSD symptoms. It has been shown that individuals with PTSD have a selective deficit in cognitive flexibility (Levy-Gigi et al., 2012, 2015; Ben-Zion et al., 2018; Haim-Nachum and Levy-Gigi, 2019), and that impaired regulatory flexibility predicted PTSD symptoms following trauma exposure (Levy-Gigi et al., 2016). However, to date, no study has tested the relative effect of cognitive compared to regulatory flexibility on PTSD symptoms. In order to provide a wider examination, we differentiate between current PTSD symptoms (experienced in the last month) and lifetime PTSD symptoms (Davidson et al., 1997). Based on existing findings, we hypothesized that both cognitive and regulatory flexibility will negatively correlate with levels of PTSD symptoms, with greater flexibility predicting reduced current and lifetime PTSD symptom.

Focusing on PTSD symptom levels rather than the dichotomy of the presence or absence of pathology is in line with the current shift to dimensional approach (Cuthbert and Kozak, 2013; for review, see Carcone and Ruocco, 2017). Such an investigation provides a wide perspective on flexibility as a possible mechanism involved in symptom development and maintenance.

## Methods

### Participants

We used G^∗^Power software to determine a sufficient sample size given an alpha of 0.05, a power of 0.90, and a medium effect size (*f*^2^ = 0.15) (Faul et al., 2007). Based on the aforementioned assumptions, we conducted *a priori* power analysis for linear multiple regression, which revealed the need for 99 participants. The estimated sample size was increased by 10% to account for potential equipment failure and to ensure high data quality. Using in-campus advertisements, we therefore recruited 109 Israeli college-student volunteers (age range = 19–39 years) who have experienced at least one traumatic event in their lifetime to participate in the study (for a detailed description of the sample, see Table 1). All participants completed a mandatory military service (time in service ranges between 24 and 36 months). To reduce confounds related to concurrent disorders, we used the *Structured Clinical Interview for DSM-5* (SCID-5-CT; First et al., 2015) to exclude participants with any psychiatric disorders other than PTSD (For descriptive statistics of individuals with as compared to without PTSD, see Supplementary Table 1). The SCID-5 was administrated by a well-trained clinical psychologist at a post-doctoral level during a face-to-face meeting at the lab; each interview lasted approximately 40 min. One participant was excluded due to depression. The investigation was conducted in accordance with the latest version of the Helsinki Declaration. The study design was reviewed by the Institutional Review Board of Bar-Ilan University. Informed consent was obtained after the nature of the procedure was fully explained. Each participant was then interviewed in a quiet room at the lab and consecutively completed the self-report questionnaires, starting with the flexibility scales followed by the trauma exposure scale and clinical measures. At the end of the study, participants were debriefed.

**TABLE 1:** Demographic characteristics and clinical measures of the participants (standard deviations in parentheses).

Variables	Mean (SD)
Age (years)	25.31 (4.59)
Female/Male (Ns)*	86/23
Education (years)	14.43 (2.16)
Single/Married (Ns)	76/33
Current PTSD	29.23 (11.34)
Lifetime PTSD	40.89 (26.56)
Cognitive Flexibility	56.29 (7.22)
Ability to Flexibly Use Coping Strategies	9.16 (2.13)
Ability to Flexibly Modulate Expression	15.27 (2.84)
Trauma Exposure	2.37 (1.61)

*N* = 109. *Independent t-test results revealed no significant differences between cognitive and regulatory flexibility levels and current and lifetime PTSD symptoms as a function of gender (all ps < 0.05). The values for Female/Male and Single/Married represent frequencies. Current PTSD scores were estimated by the PTSD checklist for the DSM-5 (PCL-5; Weathers et al., 1993), and lifetime PTSD scores were assessed using the Davidson Trauma Scale (DTS; Davidson et al., 1997); cognitive flexibility levels were measured using the Cognitive Flexibility Scale (CFS; Martin and Rubin, 1995); the ability to flexibly use coping strategies was assessed using the Perceived Ability to Cope with Trauma (PACT; Bonanno et al., 2011); and the ability to flexibly modulate expression was measured using the Flexible Regulation of Emotional Expression (FREE; Burton and Bonanno, 2016). Trauma exposure was assessed using the Traumatic Events Questionnaire (TEQ; Vrana and Lauterbach, 1994).

### Measurements

Exposure to traumatic events was measured using the *Traumatic Events Questionnaire* (TEQ; Vrana and Lauterbach, 1994), an 11-item questionnaire (Internal consistency α = 0.86, range = 1–7 events. Forty-four participants experienced one traumatic event, 46 experienced two or three events, and 19 participants experienced between four and seven events). The TEQ includes specific types of potentially traumatic events to assess lifetime exposure. Items include combat, fire/explosions, severe accidents, natural disasters, violent crime, sexual assault, abusive relationship in adulthood, physical/sextual abuse in childhood, witnessing someone being seriously injured/killed, unexpected death of a loved one, and other life-threatening situations.

The main outcome measures were the *Posttraumatic Stress Disorder Checklist for DSM-5* (PCL-5; Weathers et al., 1993), a 20-item self-report questionnaire (Internal consistency α = 0.91) corresponding to the DSM-5 symptoms of PTSD over the past month, using a 5-point scale ranging from 0 = “not at all” to 4 = “extremely.” A sample item is, “In the past month, how much were you bothered by repeated unwanted disturbing memories of the traumatic event”?; and the *Davidson Trauma Scale* (DTS; Davidson et al., 1997), in which participants are asked to record their most disturbing trauma while rating 17 items (Internal consistency α = 0.95) measuring the lifetime frequency and severity of these symptoms on a 5-point scale ranging from 0 = “not at all” to 4 = “extremely” in three clusters: intrusion, avoidance, and hyperarousal. A sample item is, “Have you had painful images, memories or thoughts of the event?”

Cognitive flexibility was measured using the *Cognitive Flexibility Scale* (CFS; Martin and Rubin, 1995), a 12-item self-report questionnaire (Internal consistency α = 0.77) that assesses the ability to communicate effectively, particularly in new situations. The CFS has three primary scales: awareness of options for one’s behavior, willingness to be flexible, and self-efficacy in being flexible. Each item is rated from 1 = “strongly disagree” to 6 = “strongly agree.” A sample item is, “I can communicate an idea in many different ways.” The total score of these sub-scales was used to indicate cognitive flexibility levels.

Regulatory Flexibility was assessed using two different questionnaires, each measured a different aspect of this flexibility: *The Flexible Regulation of Emotional Expression* (FREE; Burton and Bonanno, 2016), a 16-item questionnaire (Internal consistency α = 0.71) for measuring a person’s ability to enhance and suppress displayed emotion across an array of hypothetical contexts on a 6-point scale ranging from 1 = “not at all” to 6 = “very much.” A sample item is, “Indicate how well would you be able to be even more expressive than usual of how you are feeling: A friend wins an award for a sport that does not interest you.”; and the *Perceived Ability to Cope with Trauma* (PACT; Bonanno et al., 2011), a 20-item questionnaire (Internal consistency α = 0.91) that measures individuals’ flexibility to cope and the ability to use certain strategies and behaviors in response to events that are aversive or potentially traumatic, with two scales that measure the perceived ability to focus on processing trauma (trauma focus) and the perceived ability to focus on moving beyond the trauma (forward focus), using a 7-point scale ranging from 1 = “not at all able” to 7 = “extremely able.” A sample item is, “Rate the extent that you would be able to alter your daily routine following traumatic event if you needed to.” For each questionnaire, the overall flexibility score was used for the current study analysis.

### Data Analyses

We used IBM SPSS statistics for Windows, Version 25 for data analysis. We first used Pearson’s correlations to test associations between cognitive flexibility and regulatory flexibility measures, as well as the associations between all flexibility measures and current and lifetime PTSD symptoms. In addition, we applied a multiple linear regression analysis using flexibility measures to predict current and lifetime PTSD symptom levels. Due to the different ratio of men and women in our sample, we conducted an independent-sample *t*-test to evaluate whether there are differences between cognitive and regulatory flexibility levels and current and lifetime PTSD symptoms as a function of gender. The results revealed no significant effects of gender (all *p*s > 0.05).

## Results

We first performed zero-order correlations to test the relationship between cognitive and regulatory flexibility scores using the CFS, PACT, and FREE scales as well as their relation to current and lifetime PTSD symptoms (see Supplementary Table 2). As expected, cognitive flexibility positively correlated with regulatory flexibility scores as reported in both the PACT, *r*(107) = 0.32, *p* = 0.001 (see Supplementary Figure 1), and the FREE, *r*(107) = 0.28, *p* = 0.003 (see Supplementary Figure 2), questionnaires. Specifically, we found weak yet statistically significant correlations between participants’ cognitive flexibility scores (CFS) and regulatory flexibility scores (PACT and FREE), suggesting that flexibility is in fact two separate abilities with little overlap between them. Although the correlation between regulatory flexibility measures – the FREE and PACT, *r*(107) = 0.32, *p* = 0.001, was not statistically significant than the correlation between cognitive and regulatory flexibility measures, it crosses Cohen’s (1988) threshold of a medium-sized relation. Moreover, given that these scales are theoretically designed to measure regulatory flexibility, we referred to these measures as a relatively unified construct. In addition, aligning with our prediction, we found significant inverse correlations between the CFS, PACT, FREE and both current, *r*(107) = −0.42, *p* < 0.001, *r*(107) = −0.32, *p* = 0.001, *r*(107) = −0.29, *p* = 002, and lifetime PTSD symptoms, *r*(107) = −0.32, *p* = 0.001, *r*(107) = −0.33, *p* < 0.001, *r*(107) = −0.30, *p* = 0.002, respectively.

**FIGURE 1:**
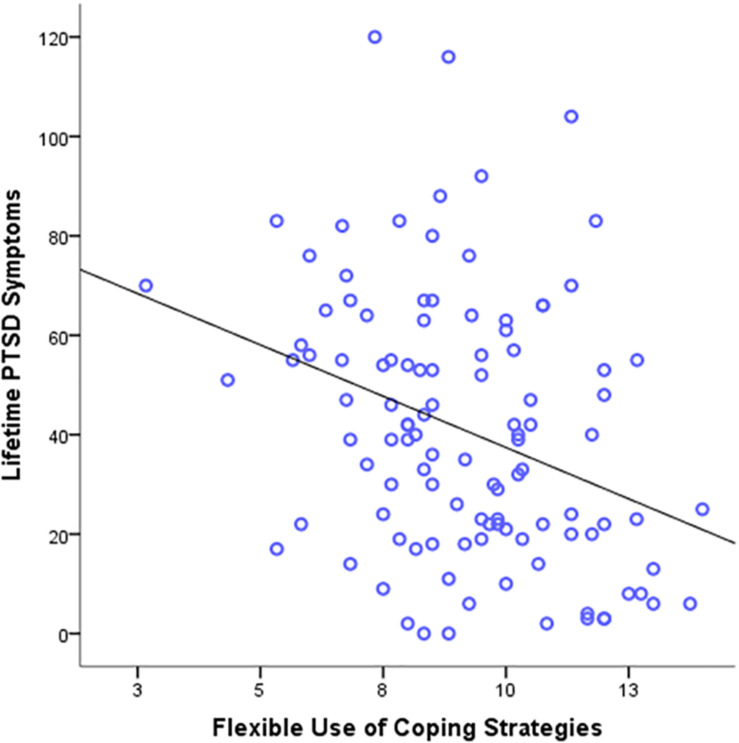
Scatter plots depicting lifetime PTSD symptoms severity as a function of flexible use of coping strategy.

We then performed a regression analysis using cognitive and regulatory flexibility levels to predict *lifetime* PTSD symptoms (Table 2). As expected, cognitive flexibility levels significantly predicted lifetime PTSD symptoms, β = −0.75, *p* = 0.03, 95% CI [−1.44, −0.06], Durbin-Watson value = 2.10 (see Supplementary Figure 3). In addition, regulatory flexibility scores in the PACT Scale also significantly predicted lifetime PTSD symptoms, β = −2.63, *p* = 0.03, 95% CI [−5.01, −0.26], Durbin-Watson value = 1.83 (Figure 1). However, in contrast to our prediction, regulatory flexibility levels as reported in the FREE Scale did not predict lifetime PTSD symptoms, β = −1.62, *p* = 0.07, 95% CI [−3.37, 0.14]. This overall pattern remained similar when we separated the two components of the FREE scale: Enhancement β = −0.11, *p* = 0.20, and Suppression β = −0.08, *p* = 0.41.

**TABLE 2:** Results of mixed regressions for the Cognitive Flexibility (CFS) and the Regulatory Flexibility (PACT, FREE) scores on both Current and Lifetime PTSD symptoms.

Dependent	Independent	*B*	*SE.B*	β	*t*	*R*^2^
variables	variables					
Current PTSD	CFS	−0.51	0.14	−0.33	−3.54***	0.23***
	PACT	−0.88	0.50	−0.17	–1.78	
	FREE	−0.59	0.37	−0.15	–1.60	
Lifetime PTSD	CFS	−0.75	0.35	−0.20	−2.15*	0.19***
	PACT	−2.63	1.19	−0.21	−2.20*	
	FREE	−1.62	0.88	−0.17	–1.83	

*p < 0.05, ***p ≤ 0.001.

Cognitive (CFS) and regulatory flexibility (PACT) also predicted lifetime PTSD symptoms when we distinguished between the frequency and intensity of these symptoms. Specifically, cognitive flexibility and regulatory flexibility scores (as reported in the PACT scale) predicted frequency: β = −0.22, *p* = 0.02, 95% CI [−0.73, −0.06]; β = −0.20, *p* = 0.04, 95% CI [−2.36, −0.03], and intensity of these symptoms: β = −0.18, *p* = 0.05, 95% CI [−0.71, 0.01]; β = −0.22, *p* = 0.02, 95% CI [−2.67, −0.20], for CFS and PACT, respectively. However, regulatory flexibility as reported in the FREE did not predict frequency or intensity of these symptoms (all *p*s > 0.05).

**FIGURE 2:**
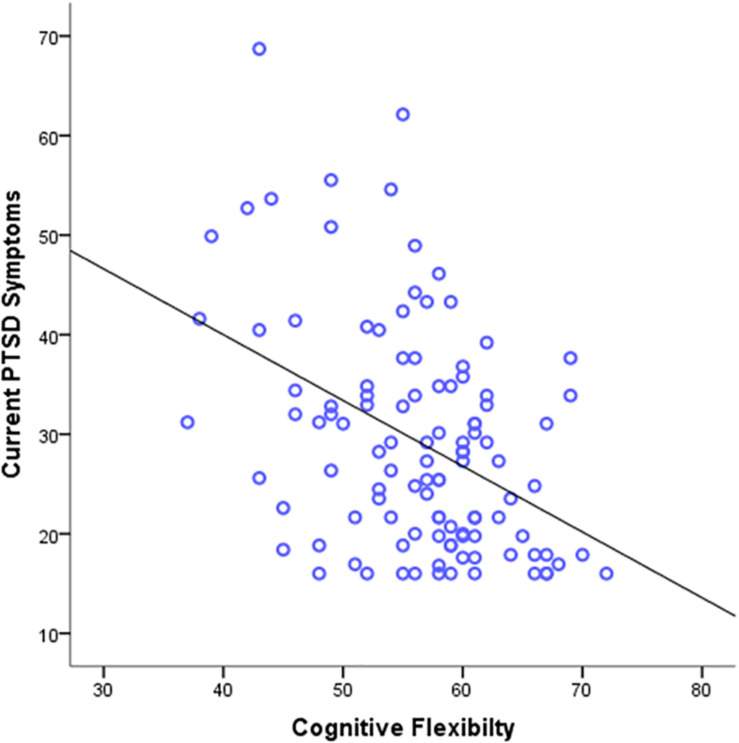
Scatter plots depicting current PTSD symptoms severity as a function of cognitive flexibility.

In a similar analysis, self-reported flexibility scores from the CFS, PACT, and FREE scales were used to predict *current* PTSD symptoms (Table 2). The results revealed that cognitive flexibility levels significantly predicted current PTSD symptoms, β = −0.51, *p* < 0.001, 95% CI [−0.80, −0.22] (Figure 2). However, in contrast with our prediction, regulatory flexibility scores from both the PACT and the FREE scales did not predict current PTSD symptoms, β = −0.88, *p* = 0.08, 95% CI [−1.87, 0.10], and β = −0.59, *p* = 0.11, 95% CI [−1.32, 0.14], respectively. When we separated the two components of the FREE scale, the results remain similar: Enhancement β = 0.10, *p* = 0.30, and Suppression β = −0.15, *p* = 0.13. Finally, the overall pattern of results remained consistent when we included age as a covariate.

## Discussion

The aim of the present study was to examine the relationship between cognitive and regulatory flexibility and their relative effects on current and lifetime PTSD symptoms. We predicted and found a weak yet significant relationship between cognitive and regulatory flexibility, further suggesting that the relationship between the different flexibility constructs is complex. Moreover, it indicates that the term flexibility refers not to one general ability but rather to at least two distinct abilities and thus, should be used with caution. These findings are consistent with previous studies exploring interactions between cognitive and emotional processes (Miller, 2010). For example, empathy, which was once thought to be one unified construct, is now typically studied as having two separate components: cognitive and emotional empathy (Shamay-Tsoory et al., 2009; Levy-Gigi and Shamay-Tsoory, 2017).

In addition, our findings show that cognitive and regulatory flexibility each have a differential effect on PTSD symptoms. Specifically, in line with previous studies, *current* PTSD symptoms were most strongly inversely associated with levels of cognitive – but not regulatory – flexibility (Ben-Zion et al., 2018; Haim-Nachum and Levy-Gigi, 2019), whereas *lifetime* PTSD symptoms were inversely associated with both cognitive and regulatory flexibility, with the latter being numerically most strongly associated with these symptoms. That is, people’s inabilities to change their thoughts and adaptively respond to different situations are more strongly related to recent PTSD symptoms, whereas people’s inabilities to use a variety of regulatory strategies are more strongly related to increased lifetime PTSD symptoms. A possible explanation for these results is that recent PTSD symptoms may be more inversely related to global beliefs about oneself and one’s abilities (in line with a Cognitive Processing Therapy framework; Held et al., 2018; for a review, see, Zalta, 2015), while lifetime PTSD symptoms may be more inversely related to the ways in which a person could or has coped (Rauch et al., 2013; Sheerin et al., 2018).

The findings bear clinical relevance, demonstrating the value of differentiating between cognitive and regulatory flexibility in predicting current and lifetime PTSD symptoms. They suggest that prevention and treatment of PTSD symptoms should be designed to address distinct constructs of flexibility, rather than increasing flexibility as one unified ability. Moreover, whereas cognitive flexibility may be an essential first line of defense for the treatment of current PTSD symptoms, improving regulatory flexibility may be important in the long-term, in order to reduce lifetime PTSD symptoms.

Importantly, our results demonstrate that the inability to shift between coping strategies, but not the inability to modulate emotion, was significantly associated with lifetime PTSD symptoms. This finding expands previous results by demonstrating that the impaired capacity to enact coping behavior (i.e., regulate *expression*, not experience, of emotions) is less dominant compared to the inability to use coping strategies in predicting not only depression and anxiety (Chen et al., 2018) but also PTSD symptoms. Together, the results emphasize the importance of distinguishing between flexibility constructs as well as between types of PTSD symptoms.

While the present study serves as a crucial first step toward understanding the relative effects of cognitive and regulatory flexibility on PTSD, several limitations must be noted. First, whilst the study highlights the role of impairments in cognitive flexibility as compared to regulatory flexibility in predicting current PTSD symptoms, future studies may aim to extend this investigation by including additional constructs of flexibility such as behavioral (Brown and Tait, 2010) and explanatory flexibility (Joseph and Gray, 2011), in order to derive a more nuanced understanding of how flexibility affects PTSD symptoms.

Moreover, in line with the current shift to a dimensional approach to psychopathology, we focused on subclinical populations rather than using dichotomous clinical definitions. While our study provides important insights which may promote the development of interventions to reduce levels of PTSD symptoms, it does not allow to differentiate between various symptom clusters. Future studies may consider recruiting a larger sample of individuals diagnosed with PTSD in order to test the relationship between cognitive and regulatory flexibility and different PTSD symptom clusters. Such investigation may be highly informative in designing specific intervention methods to improve flexibility and diminish specific PTSD symptom clusters (Pinciotti et al., 2017). For example, it is possible that increasing individuals’ cognitive flexibility will reduce intrusion symptoms as they involve re-experiencing the event despite being removed from the traumatic context. On the other hand, increasing one’s regulatory flexibility, specifically the ability to apply different coping strategies in response to contextual demands, may reduce hyperarousal and alertness and help balance extreme emotions.

In addition, women accounted for more than 3/4 of our sample. Although *t*-tests revealed no significant differences as a function of gender, future studies should recruit a more balanced sample to draw valid conclusions and avoid gender bias/ensure an equal representation.

Finally, we evaluated cognitive and regulatory flexibility solely through the use of self-report measures. While these measures are widely used and provide insights into individuals’ behaviors (Palm and Follette, 2011; Freeman et al., 2013; Myruski et al., 2017; Rodin et al., 2017), future studies may wish to include both self-report and performance-based paradigms in order to complement these findings.

In this same vein, future studies may aim to use a longitudinal design and test the predictive relationship between cognitive and regulatory flexibility with PTSD symptoms across time, to reach more conclusive results regarding causality.

In conclusion, the results of the present study serve as a first step toward differentiating the constructs of cognitive and regulatory flexibility and their interactive, yet distinct, impact on PTSD symptoms. Our findings emphasize the importance of distinguishing between flexibility constructs as well as between types of PTSD symptoms, which could in turn pave the way to designing more specific flexibility-based protocols and interventions for PTSD and promoting adaptive behavior.

## Data Availability Statement

The datasets presented in this study can be found in online repositories. The names of the repository/repositories and accession number(s) can be found in the article/Supplementary Material.

## Ethics Statement

The studies involving human participants were reviewed and approved by the IRB of Bar-Ilan University. The participants provided their written informed consent to participate in this study.

## Author Contributions

EL-G developed the concept of the study. SH-N collected the data, preformed the statistical analyses and wrote a first draft of the manuscript. EL-G edited and revised the text. All authors reviewed and confirmed the final version of the manuscript.

## Conflict of Interest

The authors declare that the research was conducted in the absence of any commercial or financial relationships that could be construed as a potential conflict of interest.
