# Supplementary figures and images for: The Tension Between Cognitive and Regulatory Flexibility and Their Associations With Current and Lifetime PTSD Symptoms
Source: Front Psychol. 2021 Feb 26;12:615289. doi: 10.3389/fpsyg.2021.615289 (PMC7959847; doi:10.3389/fpsyg.2021.615289)

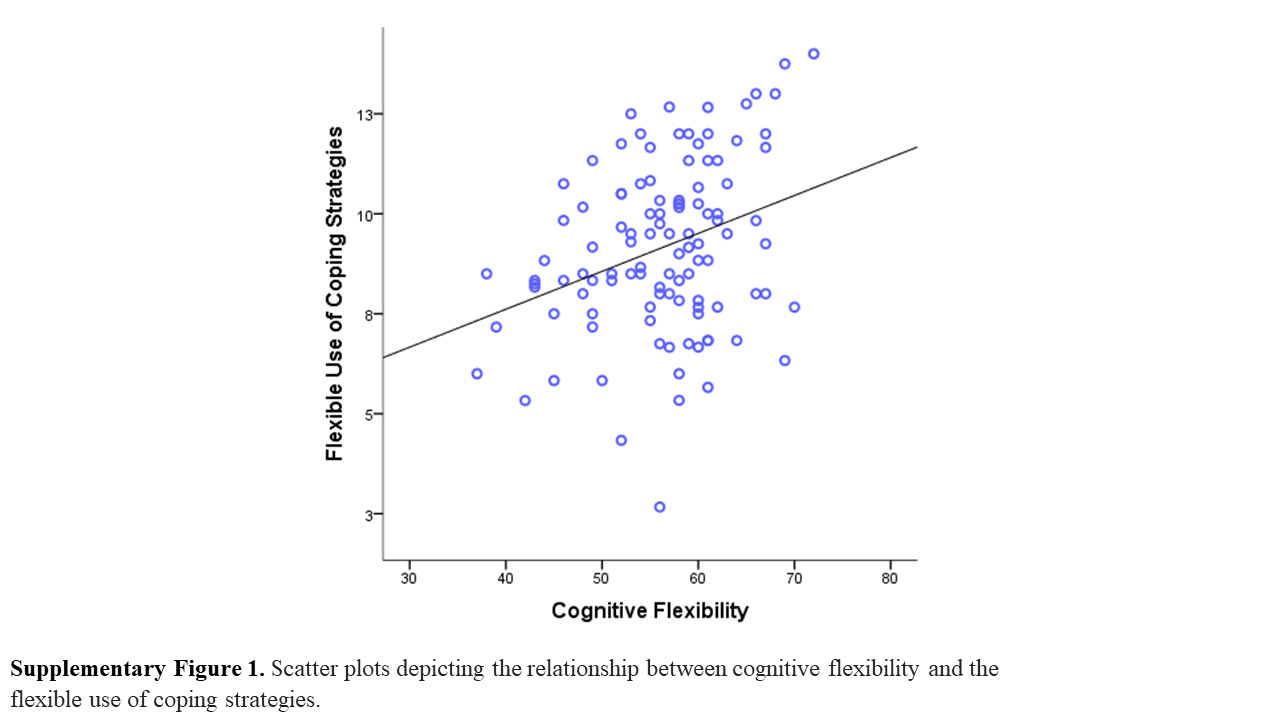

Supplement: Supplementary file 1 [file Image_1.TIF]

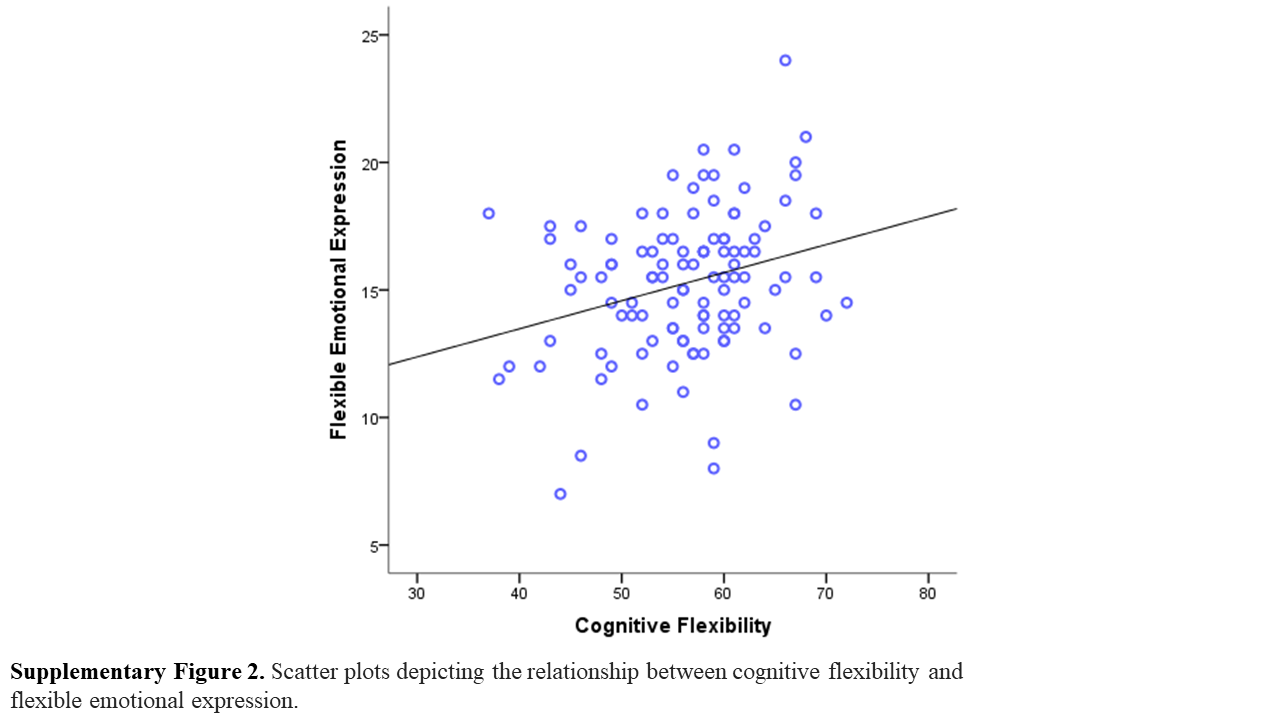

Supplement: Supplementary file 2 [file Image_2.TIF]

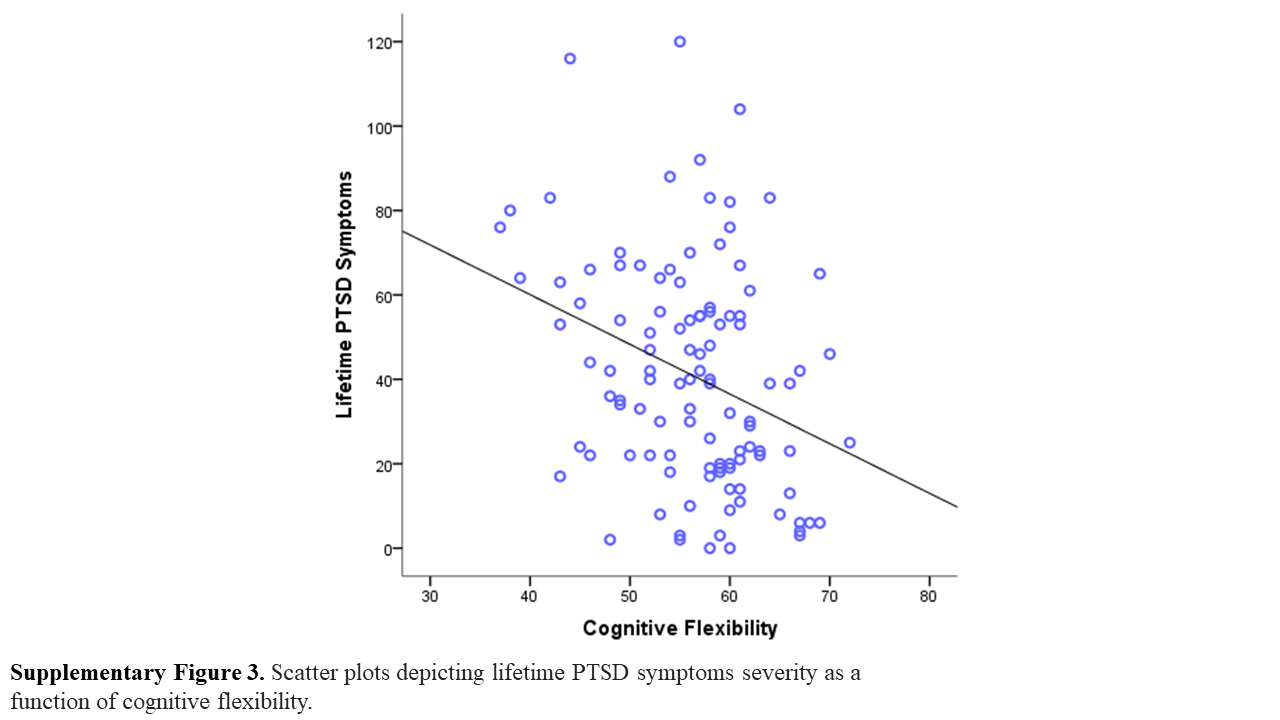

Supplement: Supplementary file 3 [file Image_3.TIF]
