# Supplementary material for: The Tension Between Cognitive and Regulatory Flexibility and Their Associations With Current and Lifetime PTSD Symptoms
Source: Front Psychol. 2021 Feb 26;12:615289. doi: 10.3389/fpsyg.2021.615289 (PMC7959847; doi:10.3389/fpsyg.2021.615289)
Supplement: Supplementary file 4 [file Table_1.docx]

| Variables | Mean (SD) | |
| --- | --- | --- |
|  | PTSD | Non-PTSD |
| N | 45 | 64 |
| Age (years) | 23.95 (4.10) | 26.12 (4.79) |
| Female/Male (Ns) | 38/7 | 48/16 |
| Education (years) | 13.98 (2.14) | 14.75 (2.13) |
| Single/Married (Ns) | 39/6 | 37/27 |
| Current PTSD | 40.05 (9.06) | 21.63 (4.56) |
| Lifetime PTSD | 55.15 (23.91) | 30.86 (23.71) |
| Cognitive Flexibility | 53.35 (7.82) | 58.36 (6.01) |
| Ability to Flexibly Use Coping Strategies | 8.53 (2.21) | 9.60 (1.97) |
| Ability to Flexibly Modulate Expression | 14.33 (2.98) | 15.93 (2.56) |
| Trauma Exposure | 2.60 (1.78) | 2.20 (1.48) |

**Supplementary Table 1**

*Comparison of demographic characteristics and clinical measures of participants with or without PTSD (standard deviations in parentheses)*

*Note.* The values for Female/Male and Single/Married represent frequencies.
