# Supplementary material for: The Tension Between Cognitive and Regulatory Flexibility and Their Associations With Current and Lifetime PTSD Symptoms
Source: Front Psychol. 2021 Feb 26;12:615289. doi: 10.3389/fpsyg.2021.615289 (PMC7959847; doi:10.3389/fpsyg.2021.615289)
Supplement: Supplementary file 5 [file Table_2.docx]

**Supplementary Table 2**

*Zero-order correlations between Cognitive Flexibility, Regulatory Flexibility (PACT, FREE), Current and Lifetime PTSD symptoms*

| Variables | 1 | 2 | 3 | 4 | 5 |
| --- | --- | --- | --- | --- | --- |
| 1. Cognitive flexibility | 1 |  |  |  |  |
| 2. PACT scores | .32*** |  |  |  |  |
| 3. FREE scores | .28** | .32*** |  |  |  |
| 4. Current PTSD | -.42*** | -.32*** | -.29** |  |  |
| 5. Lifetime PTSD | -.32*** | -.33*** | -.30** | .50*** | 1 |

***p* ≤ .01. ****p* ≤ .001*.*

*Note.* Cognitive flexibility was measured using the Cognitive Flexibility Scale (CFS). Regulatory flexibility was assessed using the Perceived Ability to Cope with Trauma (PACT) and Flexible Regulation of Emotional Expression (FREE) scales. Current PTSD scores were estimated by the PTSD checklist for the DSM-5 (PCL-5), and lifetime PTSD scores were assessed using the Davidson Trauma Scale (DTS).
